# The Significance of Apolipoprotein E Measurement in the Screening of Fetal Down Syndrome

**DOI:** 10.3390/jcm9123995

**Published:** 2020-12-10

**Authors:** Angelika Buczyńska, Iwona Sidorkiewicz, Sławomir Ławicki, Adam Krętowski, Monika Zbucka-Krętowska

**Affiliations:** 1 Clinical Research Centre, Medical University of Bialystok, 15-276 Bialystok, Poland; angelika.buczynska@umb.edu.pl (A.B.); iwona.sidorkiewicz@umb.edu.pl (I.S.); adamkretowski@wp.pl (A.K.); 2 Department of Population Medicine and Civilization Diseases Prevention, Medical University of Bialystok, 15-276 Bialystok, Poland; slawicki@umb.edu.pl; 3 Department of Endocrinology, Diabetology and Internal Medicine, Medical University of Bialystok, 15-276 Bialystok, Poland; 4 Department of Gynecological Endocrinology and Adolescent Gynecology, Medical University of Bialystok, 15-276 Bialystok, Poland

**Keywords:** Down syndrome, prenatal diagnosis, apolipoprotein E

## Abstract

Prenatal screening for Down syndrome (DS) is based on both noninvasive and invasive methods. Noninvasive, cell-free fetal DNA genetic tests are expensive, whereas biochemical methods remain imprecise. Amniocentesis is the most frequently used invasive diagnosis procedure, characterized by 99.8% diagnostic efficiency and less than 1% risk of miscarriage. The aim of this study was to evaluate the screening value of apolipoprotein E (ApoE) as a potential noninvasive biomarker for prenatal DS assessment. This study was conducted on a group of female patients who decided to undergo routine amniocentesis between the 15th and 18th week of pregnancy at the Department of Reproduction and Gynecological Endocrinology of the Medical University of Bialystok, Poland. For the purpose of this study, 20 women with DS fetuses were selected as the study group, and 20 healthy pregnant women with euploid fetus karyotypes as the control group. The plasma levels of ApoE were significantly higher in the study group compared to healthy subjects (*p* < 0.05). The area under the receiver operating characteristic (ROC) curve was 0.978 (*p* < 0.001), with the cut-off set to 1.37 mg/mL, which was characterized by 80% of sensitivity and 100% of specificity. The high sensitivity and specificity demonstrate the screening utility of maternal ApoE concentration in prenatal fetal DS screening.

## 1. Introduction

Trisomy 21, also known as Down syndrome (DS), is one of the most frequently occurring chromosomal disorders worldwide [1,2]. The disease affects 1 in every 787 live births and is characterized by the abnormal division of genetic material resulting in an additional chromosome 21 or its part [3]. Trisomy 21 is the major cause of DS, accounting for about 95% of cases [4]. This disease results in unequal distribution of DNA material and metabolic pathway dysfunction, including lipid metabolism disturbances, increased oxidative stress, mitochondrial dysfunction, and tau phosphorylation [5]. Maternal age above 35 years and the occurrence of balanced translocation in one of the parents are the main risk factors.

Apolipoprotein E (ApoE), a glycoprotein with a linear polypeptide chain rich in arginine, is a hydrophilic component of high-density lipoprotein (HDL), very low-density lipoprotein (VLDL), lipoproteins, and chylomicrons [6]. It is mainly synthesized in the cell periphery of hepatocytes, but also in macrophages, astrocytes, lungs, kidneys, spleens, and muscle cells, with the highest expression in the liver and brain. In the brain, the main supply of ApoE is in the blood–brain barrier, and it is observed in the cerebrospinal fluid at concentrations of ~5 mg/L; it is mainly produced by astrocytes, neurons, and damaged microglia [6,7]. The maturation of the fetus is dependent on the undisrupted development of the nervous system during its growth. Approximately 23% of lipids are accumulated in the brain, particularly in neurons and astrocytes. During the first few weeks of gestation, the developing fetus mainly uses maternal cholesterol. Fetal cholesterol and apolipoproteins (ApoA1, ApoE, and ApoB), together with HDL, LDL, or VLDL are crucial for moderating embryonic signaling pathways [8]. Therefore, ApoE is considered to be a promoter of myelination, synaptogenesis, and other processes related to neurodevelopment that involve lipids.

Nowadays, prenatal screening of DS is based on both noninvasive methods, which estimate the risk of DS-affected pregnancy, and invasive techniques, which verify the presence of chromosomal aberrations. Serum screening and ultrasounds are intended to identify women with pregnancies at high risk of chromosomal abnormalities. However, diagnostic testing is indicated in international guidelines, mainly focusing on individual risk assessment based on historical, biochemical, and biophysical variables [9]. Amniocentesis is the most frequently used invasive procedure, which has a diagnostic efficacy of 99.8% and poses less than 1% risk of miscarriage [10,11,12]. The discovery of genetic testing using free fetal DNA (ffDNA), whose concentration can be measured in maternal peripheral blood, was a significant breakthrough in noninvasive screening. In addition to the 0.5% false-positive rate, this technique is still comparatively expensive [13,14,15,16,17]. Therefore, it is important to find a cost-effective and noninvasive screening biomarker with high sensitivity and specificity that would provide indisputable benefits, subsequently reducing the number of incorrect indications for amniocentesis diagnosis. Moreover, more comprehensive knowledge of DS pathogenesis, including the understanding of metabolic pathway alterations, may introduce new treatment targets and improve patients’ quality of life.

The aim of the study was to evaluate the screening usefulness of maternal ApoE measurement as a potential noninvasive marker in prenatal diagnostics of DS.

## 2. Experimental Section

The study and control groups consisted of women who underwent routine amniocentesis between the 15th and 18th weeks of gestation at the Department of Reproduction and Gynecological Endocrinology of the Medical University of Bialystok, Poland. The indication for amniocentesis was an increased risk of chromosomal aberrations in noninvasive prenatal screening or patient age above 35 years. Exclusion criteria were as follows: chronic or acute diseases, hormonal treatment, anti-inflammatory treatment, high-risk pregnancy, or preterm delivery in the patient’s medical history. All participants were informed about potential risks prior to the procedure and received relevant information regarding the study. Study participants were matched according to age, ethnicity, socioeconomic status, the course of pregnancy, body mass index (BMI), and the number of pregnancies with marked episodes of pregnancy pathology. The patient recruitment period started in 2017 and lasted 2 years. Following karyotype test result analysis, 20 women carrying fetuses with DS and 20 women with euploid (non-DS) fetuses were enrolled in the study. All patients were submitted to amniocentesis within a determined period, then randomized. An adequate sample size to detect a difference was demonstrated using power analysis [18]. Venous blood (5.5 mL) was obtained from participants at the day of amniocentesis, centrifuged, and plasma was subsequently separated and frozen at −80 °C.

Plasma ApoE concentration was determined using an enzyme-linked immunosorbent assay (ELISA) (ELISA Kit for apolipoprotein E (ApoE); Cloud-Clone Corp., Wuhan, Hubei 430056, China, SEA704Hu) according to the manufacturer’s protocol and observing the principles of internal laboratory control for the performed determinations. Samples and controls were measured in the same run using the blind analysis method. Duplicate samples were assessed and the average of the two results was calculated [19]. Statistical analyses were performed using Statistica 13.3 (StatSoft, Tibco Software Inc., Palo Alto, CA, USA). During the analysis, the normality of data distribution was demonstrated (*p* > 0.05). Thus, the groups were compared using a parametric two-way ANOVA test; *p* < 0.05 was considered statistically significant. In addition, the receiver operating characteristic (ROC) curves were determined using a medical package included in the Statistica program. Diagnostic sensitivity and specificity were calculated using a cut-off value that was calculated by the Youden’s index (as a criterion for selecting the optimum cut-off point) [20].

The procedures were approved by the Local Ethics Committee of the Medical University of Bialystok, Poland, and written informed consent was obtained from each participant (R-I-002/36/2014).

## 3. Results

In this research, the first participant was entered on 1 January 2015. The last participant was randomized on 12 April 2019, at the end of the trial. A total value of 100 pregnant women were screened, and 40 were randomized and enrolled for the subsequent analysis.

### Statistical Analyses

**Figure 1:**
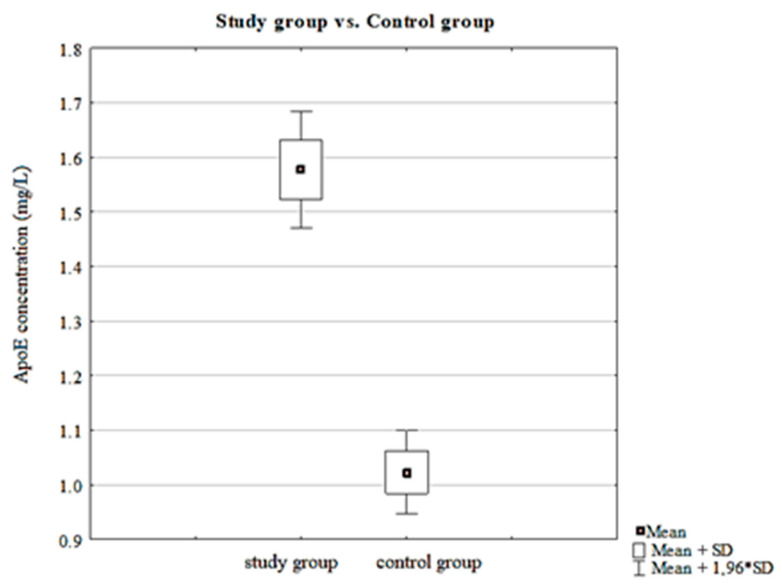
Concentration of apolipoprotein E (ApoE) measurements in study vs. control group. SD, standard deviation.

Basic statistics that were measured in the study, control, and total group, such as average ApoE concentration values, minimum and maximum values, and standard error, are presented in Table 1. ApoE concentrations were significantly higher in the study group compared to healthy subjects (*p* < 0.001). The comparison of ApoE concentrations measured in the study vs. control group is presented in Figure 1.

**Figure 2:**
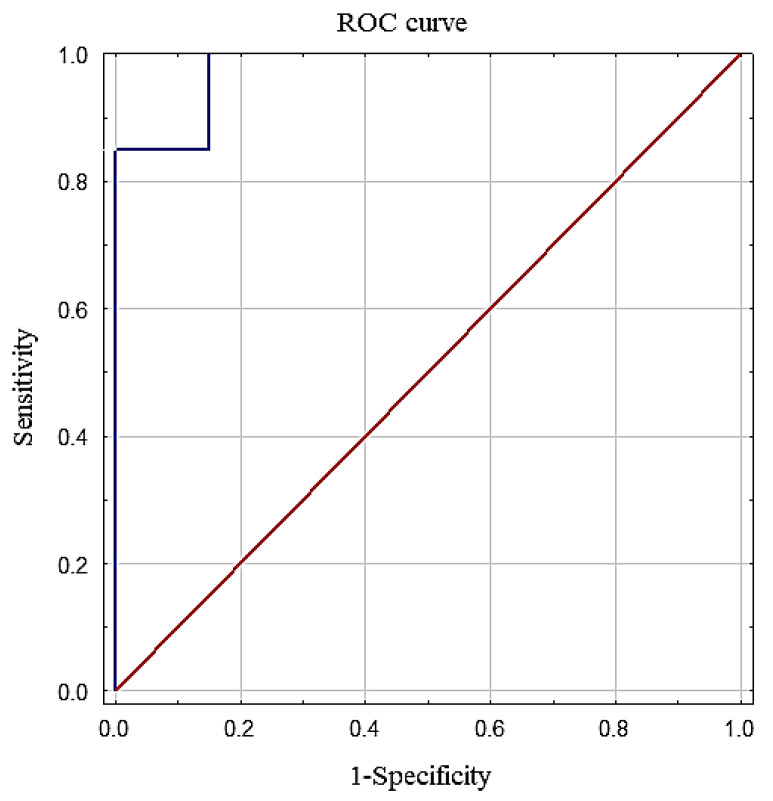
Receiver operating characteristic (ROC) curve for ApoE in Down syndrome (DS) screening.

To determine the diagnostic utility of the ApoE test, the ROC curve was calculated as an illustration of the relationship between sensitivity and specificity (Figure 2). The cut-off point was set at 0.85 using Youden’s index, simultaneously establishing the diagnostic norm of ApoE as 1.37 mg/L. The sensitivity, accuracy, specificity, and positive and negative predictive values (PPV and NPV, respectively) are presented in Table 2.

To evaluate the clinical applicability of ApoE as a prenatal screening tool, the area under the ROC curve (AUC) was evaluated. The AUC value was 0.978, which was significantly higher in comparison to AUC 0.5, which is the threshold of the diagnostic usefulness of a test (*p* < 0.001).

## 4. Discussion

Despite DS being the most common chromosomal aberration occurring in all races, the pathological process, as well as the incorrect division of DNA material, are not yet fully understood [21,22,23]. It has been demonstrated that pregnant women with a DS fetus suffer from lipid disorders related to disturbed cholesterol metabolism and lipid transport; incorrect distribution of VLDL, LDL, and lipid peroxidation [4]; and altered concentration of sphingolipids, which leads to improper myelination of fetal neurons [24]. These patients also show insufficient endothelial function, which is related to the inflammation process, oxidative stress, and dysregulated lipid metabolism, which may result in insufficient cell division [25].

Screening tests are used to survey a population by measuring a specific marker to define screening cut-off levels, with subsequent identification of a high-risk group for a particular disorder [26]. Multiple screening tests are used, involving the combination of a few biochemical tests, usually combined with maternal age or an ultrasound examination, to estimate the risk of DS occurrence [27]. To compare the diagnostic efficiency, the combination of ultrasound examination with pregnancy-associated plasma protein A (PAPP-A) and serum-free human chorionic gonadotropin (B-HCG) measurement, using a 5% screen-positive rate, allows for the detection of 82–87% DS pregnancies [28]. Increased maternal serum ApoE concentration in DS-affected pregnancies has been previously explored, but the diagnostic utility of this marker has not been comprehensively illustrated [27].

The first attempt to evaluate the influence of ApoE on adolescent neurodevelopment was made by Tratnik et al., who conducted research on the association between prenatal exposure to mercury (Hg) and child neurodevelopment, while considering genetic ApoE polymorphism. The study revealed that the presence of the APOE ε4 allele combined with Hg exposure resulted in a decline in cognitive performance in the studied children [29]. Pinto et al. demonstrated that women carrying a fetus with DS may display impaired lipid metabolism [30]. Pranami et al. demonstrated that women carrying the APOE ε4 allele and having increased cholesterol levels have impaired microcirculation in capillaries, which may cause atherosclerosis of the microcirculation vessels surrounding ovarian follicles that may result in incorrect meiotic division 2, indirectly leading to DS [31].

In our study, plasma ApoE levels were considered low, but comparable to those found in the study by Kaneva et al., where the shift in plasma ApoE toward lower levels in European residents as a result of specific features of lipid metabolism was demonstrated [32]. We also demonstrated the screening utility of maternal serum ApoE measurement as a potential noninvasive marker of DS. Comparing this result with the commonly used biochemical markers, the screening usefulness of the test was much higher (AUC = 0.978) compared to PAPP-A (AUC = 0.7771) and B-HCG (AUC = 0.6682) or combined PAPP-A + B-HCG (AUC = 0.8533) measurements [33]. Our results indicate that ApoE concentration could be added in a combined test screening approach or proposed as an alternative examination. However, further evaluation with subsequent data validation with ultrasound tests and combined screening tests using a larger cohort is required.

The study performed by Rindler et al. identified the placental ApoE synthesis as the major maternal lipid profile modifier during pregnancy. ApoE has been suggested to play a supportive role in regulating maternal and fetal homeostasis [34,35,36]. ApoE may also balance the oxidative and antioxidative processes through LDL oxidation inhibition and methylation reduction [37]. The elevated levels of oxidative stress markers were observed in DS fetuses, as well as in DS pregnancies. Referring to the result above and that obtained by Melhem et al. where the secretion pattern demonstrated a predominant maternal orientation [37], it can be concluded that maternal ApoE synthesis emphasizes its pleiotropic role in preventing fetal abnormalities [38,39]. Thus, preconception ApoE screening in women may be of clinical importance in the prediction of fetus health complications.

In our study, women with a confirmed DS pregnancy had significantly higher plasma ApoE concentrations compared with healthy subjects. The traceability of commonly used biochemical noninvasive tests can be characterized by PAPP-A tests, with sensitivity estimated at 90% with 5% false-positive results, and the triple test (combination of three markers: free β chorionic gonadotropin, α-fetoproteins, and unconjugated estriol), whose sensitivity is estimated at 60–70% [9,10,11,12,13,14]. Screening based on Caucasian reference ranges has a detection rate of 86.8% for contingent first trimester screening, 76.2% for second trimester screening, and 83.8% for their combination. However, first trimester screening had a higher false-positive rate compared to second trimester screening (13.7% vs. 7.7%) [40]. Regarding ApoE, the sensitivity, accuracy, specificity, and PPV and NPV were 80%, 82%, 100%, 83%, and 100%, respectively. The diagnostic power of the test was proven by the determination of an AUC of 0.978. Introducing maternal ApoE measurement to the methods commonly used in DS risk assessment may increase the sensitivity and specificity of noninvasive prenatal screening. However, the present investigation is a preliminary study, and further research on a larger cohort is required.

## 5. Conclusions

Our study demonstrated the relationship between maternal ApoE and fetal DS occurrence, and showed that ApoE can be used as a predictive marker of this disease, but further studies are required. The discovery of dysregulated metabolic pathways could lead to the establishment of new diagnostic targets, which may enable optimal early diagnosis, resulting in improved therapy application and enhanced quality of life.

## Figures and Tables

**Table 1:** Basic statistics of plasma ApoE measurement (data presented in mg/L).

Parameter	Study Group (*n* = 20)	Control Group (*n* = 20)	Total Group (*n* = 40)
Minimum value	1.27	0.66	0.66
Maximum value	2.18	1.31	2.18
Mean	1.57	1.02	1.3
SD	0.25	0.18	0.35

**Table 2:** Statistical parameters of ApoE measurement.

Parameter	Sensitivity	Accuracy	PPV	NPV	Specificity
ApoE (cut-off point = 1.37 mg/L)	80%	82%	100%	83%	100%

NPV, negative predictive value; PPV, positive predictive value.
